# Laplacian reconstructive network for guided thermal super-resolution

**DOI:** 10.1038/s41598-026-36027-x

**Published:** 2026-01-18

**Authors:** Aditya Kasliwal, Ishaan Gakhar, Aryan Kamani, Pratinav Seth, Ujjwal Verma

**Affiliations:** Manipal Institute of Technology, Manipal Academy of Higher Education, Manipal, India

**Keywords:** Electrical and electronic engineering, Mathematics and computing

## Abstract

In the last few years, the fusion of multi-modal data has been widely studied for various applications such as robotics, gesture recognition, and autonomous navigation. Indeed, high-quality visual sensors are expensive, and consumer-grade sensors produce low-resolution images. Researchers have developed methods to combine RGB colour images with non-visual data, such as thermal, to overcome this limitation to improve resolution. Fusing multiple modalities to produce visually appealing, high-resolution images often requires dense models with millions of parameters and a heavy computational load, which is commonly attributed to the intricate architecture of the model. We propose LapGSR, a multimodal, lightweight, generative model incorporating Laplacian image pyramids for guided thermal super-resolution. This approach uses a Laplacian Pyramid on RGB colour images to extract vital edge information, which is then used to bypass heavy feature map computation in the higher layers of the model in tandem with a combined pixel and adversarial loss. LapGSR preserves the spatial and structural details of the image while also being efficient and compact. This results in a model with significantly fewer parameters than other SOTA models while demonstrating excellent results on two cross-domain datasets viz. ULB17-VT and VGTSR datasets.

## Introduction

Super-resolution methods have applications in various domains, including digital imagery, photography, robotics, autonomous navigation, surveillance, and security^1–3^. These techniques aim to enhance the resolution of low-quality images, enabling the extraction of finer details and improved visual quality from lower-resolution source data. Recently, non-visual imagery sensors have gained prominence in various applications due to their ability to capture information beyond the visible spectrum. These sensors, such as thermal cameras, provide crucial data in challenging environments, allowing the detection of heat signatures and other phenomena not perceptible to the human eye. However, thermal images typically have low resolution, and acquiring high-resolution non-visual sensors is expensive. To address this challenge, researchers have delved into Guided Thermal Super Resolution, a field focused on enhancing the resolution of thermal images using RGB images as a guide. With the advent of deep learning, guided super-resolution methods have become increasingly effective, reducing the reliance on expensive, high-quality sensors.

**Fig. 1:**
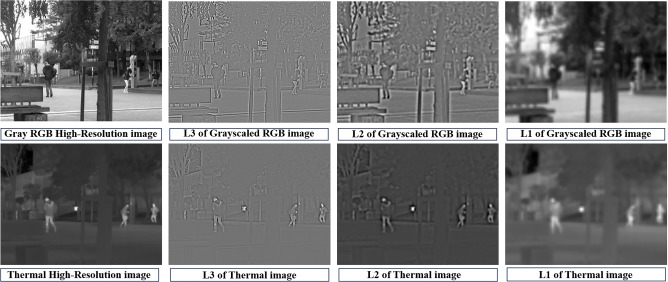
Laplacian Pyramid Visualization. The first row is the Laplacian pyramid of a grayscaled RGB image with two levels and the residual at the end. The second row contains the Laplacian pyramid of a thermal image with two levels and a residual at the end. These images have been taken from the ULB17-VT dataset^4^.

Integrating data from multiple sensors, such as RGB and thermal, can gain more comprehensive insights and enhance performance. The task of enhancing resolution in non-visual data while preserving content remains a persistent challenge^5^. We experiment with cross-domain datasets, namely the ULB17-VT^4^ dataset, which is captured by a handheld camera, and the VGTSR dataset^6^, captured by a UAV (Unmanned Aerial Vehicle) platform.

To address these issues with a streamlined approach, we introduce LapGSR. LapGSR addresses the challenges of enhancing the resolution of low-quality thermal images by leveraging information from high-resolution RGB images. Our model combines traditional computer vision techniques like Laplacian pyramids with modern deep learning methods for feature extraction. Our framework introduces key components for handling texture, illumination, and other critical features extracted from RGB inputs, ultimately enhancing the resolution of low-quality thermal images. Its architecture incorporates a lightweight, generative neural network with cascading residual blocks, providing robustness against misalignment while ensuring computational efficiency.

Our inspiration for employing Laplacian pyramids stems from their effectiveness in capturing similarities in edge maps between RGB and high-resolution thermal images. As visualized in Fig. 1, the structural features, including texture, intensity, and spatial patterns, are notably congruent within the Laplacian pyramid layers. This observation motivates our use of Laplacian pyramids for feature extraction. We utilize the Laplacian pyramid of our guiding image (RGB) to reconstruct the Laplacian pyramid of the target (thermal) image. We draw upon the Laplacian pyramid technique introduced in prior work^7^, initially devised for addressing image-to-image translation tasks encompassing variations in seasons and lighting conditions. Our proposed model allows for Guided Thermal Super Resolution in scenarios involving alignment and misalignment via the employment of residual blocks in the High Transformation Branch. Our proposed model carries out major feature map transformation in higher layers compared to their model’s lowest layers. Through our novel architecture, we achieve adaptive refinement of image structures, resulting in superior image quality. Our contributions extend across various domains, as our model preserves fine details in generated images and has fewer trainable parameters than other SOTA models, making it suitable for real-time applications where image quality is critical. Moreover, the model’s adaptability to misaligned thermal images is a significant advantage, ensuring its effectiveness in diverse scenarios. Our approach combines traditional computer vision with modern deep learning techniques, achieving state-of-the-art outcomes with fewer parameters compared to recent models in both aligned and misaligned scenarios. This highlights its effectiveness and potential influence in the super-resolution domain. The main contributions of our proposed method are:

- Incorporating the Laplacian image pyramid, a classical computer vision technique, alongside contemporary deep learning approaches for feature extraction, our contribution effectively restores high-resolution images with high visual fidelity and accuracy. This approach allows us to reduce the reliance on a large number of convolutional layers.
- A lightweight model architecture that delivers consistent performance on the VGTSR dataset and state-of-the-art performance on the ULB17-VT dataset, with 90% and 50% fewer trainable parameters than the SOTA models.
- A Multi-Objective Loss that incorporates adversarial loss to address and mitigate the trade-off between PSNR and SSIM
- Robustness against misaligned RGB-Thermal pairs, a situation often encountered in real-world scenarios, making our model suitable for deployment.

## Related works

Super Resolution finds applications in security systems, robotic vision, self-driving algorithms, etc. Existing approaches include dictionary-based methods^8,9^ and learning-based methods. A subtask of Super Resolution is Guided Thermal Super Resolution, which involves using a High-Resolution RGB image as a ‘guide’ for super-resolving the low-resolution thermal image. Thermal Super Resolution finds applications in security and surveillance systems, agriculture, autonomous navigation, etc.

### CNNs for super resolution

Convolutional Neural Networks (CNNs) for Super Resolution have become increasingly prevalent over the past decade. Their ability to learn complex relationships and hierarchical features, which assist in end-to-end mapping, is the primary reason for their superiority over previous methods. CNNs for Super Resolution were introduced through SRCNN^10^, which learned end-to-end mapping using convolutional layers and achieved superior performance by directly predicting high-resolution patches from low-resolution inputs. VDSR^11^ pioneered a very deep network for Super Resolution. VDSR’s novelty lies in its deep architecture with residual connections, effectively mitigating the vanishing gradient problem and enhancing learning at the cost of heavy computation. Using a generator-discriminator setup, SRGAN produced images with improved perceptual quality. Enhanced Deep Super-Resolution (EDSR)^12^ emphasized depth and width in network design. Its architecture optimization achieved SOTA performance with fewer parameters. However, the robustness of their model is limited since the results are only based on the validation set. ESRGAN^13^ set new benchmark in visual fidelity, which incorporated perceptual loss, adversarial loss, and a new architecture to enhance SR quality and realism. However, ESRGAN’s complex architecture includes dense layers, contributing to its high computational demand during the training and inference phases. The model’s ability to generate highly realistic and detailed images comes at the cost of increased computational resources. The use of GANs in image processing has increased over the past decade due to their ability to generate hyper-realistic images. Their implementation in super-resolution methods has improved the perceptual quality manifold. Laplacian pyramids and transpose convolution for upscaling the coarse-resolution feature maps were first used by^14^. State-of-the-art results on Guided Depth Super Resolution were achieved by^15^ by combining guided anisotropic diffusion with a deep convolutional network.

### Laplacian-Pyramid-based Super Resolution

The layers of Laplacian Image Pyramids have been used for super-resolution due to the ability of lower layers to preserve edges and the overall structural integrity of the original image while still ensuring a lightweight model with fast inference times.^7^ used a similar Laplacian Pyramid network for image-to-image translation from day-to-night or summer-to-winter. LapSRN^14^ used the layers to progressively reconstruct the sub-band residuals of high-resolution images at multiple pyramid levels. However, the reconstruction process in LapSRN involves transpose convolutions, which introduce extra parameters for the reconstruction module. Sharing parameters at every level using the same network^16^ allowed the parameter number to be independent of the upsampling scale; hence, one single set of parameters is required to construct the network with multiple pyramid levels.

### Guided thermal super resolution

Guided Thermal Super Resolution (GTSR) has been gaining relevance due to the many feasible methods proposed in recent years. High-resolution capturing RGB sensors are more economical as compared to IR sensors, and the fusion of these modalities yields a better qualitative and quantitative result than single image thermal super-resolution^17^. To increase the robustness of GTSR models^18^, incorporated a feature-alignment loss along with a misalignment map alignment block and achieved SOTA results for GTSR on unaligned datasets. Multiple U-Net backbones were used in CoReFusion^19^ to merge multiple modalities while still demonstrating comparable results for cases of missing modalities. However, the model utilizes 20M trainable parameters, owing to the two ResNet-34 encoders used for feature extraction. Self-supervised learning using a transformer to produce SOTA results without artefacts was introduced in^20^. Using two subnetworks for feature extraction from Laplacian pyramids and an attention fusion module^21^, set new qualitative and quantitative results benchmarks. Super-resolution for remote sensing applications where the data from satellite images is fused to generate HR images was also explored by^22^. They present findings across various subdomains but do not incorporate any datasets we have utilized. Their work enhances depth, DEM, and thermal imagery resolution. Of these, only the depth imagery originates from a handheld device (Middlebury and NYUv2 datasets), with the remaining datasets acquired through satellite remote sensing. In contrast, our study utilizes datasets from both handheld devices and UAVs for thermal imaging.

## Methodology

**Fig. 2:**
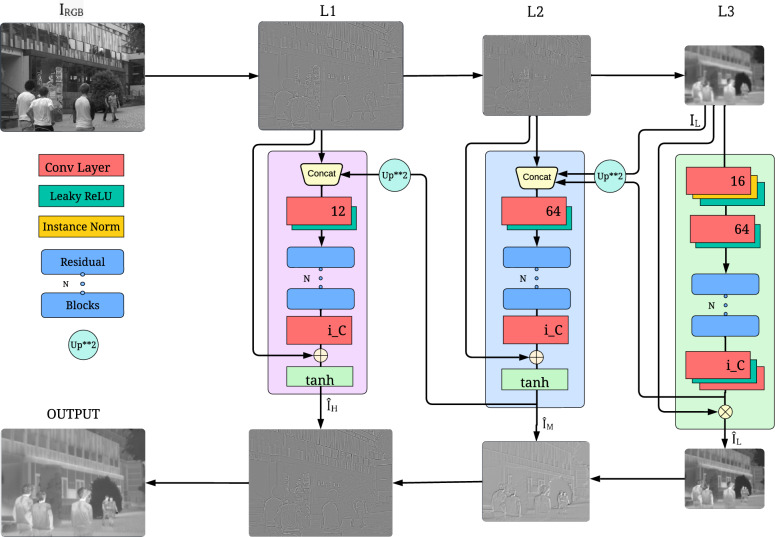
Proposed LapGSR model architecture. $I_{RGB}$ refers to the grayscaled high-resolution RGB image from the ULB17-VT dataset. The Green highlight represents the LTB, the Blue represents the MTB, and the Pink represents the HTB. The given figure is for Pyramid of Depth 2; L1, L2, and L3 represent the layers of the modified Laplacian pyramid, where the lower-resolution thermal image replaces the residual. Here, ’i_C’ refers to the number of channels of the low-resolution thermal image, which is 3 for the VGTSR dataset and 1 for the ULB17-VT dataset. Similarly, ’Up**2’ refers to bicubic upsampling by a factor of 2. The significance of each layer and relevant details are explained in Section 3.

We propose LapGSR, a lightweight, generative, robust, multimodal architecture utilizing Laplacian Pyramids for Guided Thermal Super Resolution. Our lightweight model faithfully reconstructs thermal images by the explicit edge guidance provided by the Laplacian decomposition of the RGB image (Fig. 1). As depicted in Fig. 2, we decompose the RGB image into a Laplacian pyramid of 3 levels (L3, L2, L1) and replace the last layer (residual, L1) with the low-resolution thermal image ($I_L$). LapGSR employs 3 branches responsible for feature extraction and translation from each layer of the Laplacian pyramid, namely the Lower Transformation Branch, Middle Transformation Branch, and Higher Transformation Branch. Furthermore, a brief description of the discriminator architecture is also provided in this section. Our model suits the SR factor of only 4x. To our knowledge, the GSR domain requires SR of 4x and 8x, and super-resolving by 8x would require another branch.

### Lower transformation branch

The Lower Transformation Branch (LTB) distils fundamental features such as luminance, texture, and illumination from the low-resolution thermal image. The LTB consists of a convolutional layer, followed by instance normalization and a leaky ReLU activation function is applied to prevent the vanishing gradient problem often encountered in deep networks.

After the initial feature extraction, the LTB employs an additional convolutional layer and leaky ReLU activation, followed by a series of residual blocks comprising a convolutional layer, leaky ReLU, another similar convolutional layer, and a skip connection.

The culmination of the LTB’s processing is the generation of a feature map, denoted as $\hat{I}_L$, which is the lowest layer of the translated pyramid and is also upsampled and combined with the second last layer (L2) of the branch. This enriched feature map is subsequently fed into the Middle Transformation Branch. Finally, the output of the residual blocks is multiplied with L1 to yield the final product of the LTB. By integrating the low-level details extracted by the LTB with the higher-level features, the network can construct a more comprehensive feature representation, setting the stage for advanced processing in subsequent network stages. The LTB processing can be summarized mathematically as:

1 $$\begin{aligned} \hat{I}_L = \text {LTB}(L_3) \end{aligned}$$

where $\hat{I}_L$ represents the output of the LTB.

2 $$\begin{aligned} \text {LTB}(x) = f_{\text {res}}^N(\text {LeakyReLU}(\text {Conv}(\text {LeakyReLU}(\text {InstanceNorm}(\text {Conv}(x)))))) \end{aligned}$$

where *x* represents the input to the LTB, $\text {Conv}$ denotes convolutional operations, $\text {InstanceNorm}$ is instance normalization, $\text {LeakyReLU}$ is the leaky ReLU activation function, and $f_{\text {res}}^N$ represents *N* residual blocks.

3 $$\begin{aligned} {I}_M = L_2 \oplus (\text {Upsample}^2(\hat{I}_L)) \end{aligned}$$

where ${I}_M$ is the input to the MTB, $\hat{I}_L$ is the output from the LTB, $\text {Upsample}^2$ refers to 2$\times$ upsampling, and $\oplus$ represents the concatenation operation.

### Middle transformation branch

The Middle Transformation Branch (MTB) serves as a critical component in our network, bridging the low-level feature processing conducted by the Lower Transformation Branch (LTB) and the high-level abstractions required for subsequent tasks. The MTB is tasked with refining and transforming the concatenated feature maps received from the LTB. It also doubles as a realignment module, further demonstrating the robustness of our model against misaligned images. The accentuated edge information and fine details derived from Laplacian pyramids (L2) help to bypass heavy feature map computation. These derived details allow the model to avoid an excessive number of parameters.

The MTB starts with a convolutional layer to extract features accurately without altering the input map’s spatial dimensions followed by leaky ReLU. Given that the proposed model has a more refined architecture with fewer convolutional layers than other advanced models, leaky ReLU is employed to prevent the vanishing gradient problem in this streamlined model. The MTB consist of several residual blocks. A final convolutional layer follows the residual blocks, further refining the feature maps for the activation phase.

The fusion of the low-level ($\hat{I}_L$) and intermediate-level features (L2) is then passed through a tanh activation. The output from the tanh activation ($\hat{I}_M$) encapsulates a balanced and normalized feature representation, setting the stage for the final transformation tasks within the network’s pipeline. The tanh activation is only applied at the end of MTB and HTB since major spatial transformations primarily occur in these branches. The LTB is responsible for extracting minimal features necessary for combining with upper branches and hence has no non-linearity activation.

The middle transformation branch is responsible for extracting spatial and structural information by processing and refining the joint feature maps of the LTB and the L2 layer of the pyramid. This advanced feature representation is crucial for the network’s subsequent stages, where such high-level conceptualizations of the image are vital for advanced visual tasks. This contributes to the faithful and perceptually appealing reconstruction of the final image. The output of the MTB ($\hat{I}_M$) acts as the intermediate layer of the translated pyramids. It is 2x upsampled using bicubic interpolation and concatenated with L3, where it is subsequently passed into the High Transformation Branch.

4 $$\begin{aligned} \hat{I}_M = \text {MTB}(I_{M}) \end{aligned}$$

where $\hat{I}_M$ represents the output of the Middle Transformation Branch and $I_{M}$ denotes the input to the Middle Transformation Branch consisting of concatenated feature maps from LTB and L2.

5 $$\begin{aligned} \text {MTB}(x) = \tanh (\text {Conv}(f_{\text {res}}^{N}(\text {LeakyReLU}(\text {Conv}(x))))) \end{aligned}$$

where *x* is the input feature map and $f_{\text {res}}^{N}$ denotes N residual blocks.

6 $$\begin{aligned} I_{H} = L_3 \oplus (\text {Upsample}(\hat{I}_{M})) \end{aligned}$$

where $I_{H}$ represents the input to the HTB, $\hat{I}_{M}$ is the output from the MTB, $\oplus$ represents the concatenation operation, and Upsample refers to 2x bicubic upsampling.

### High transformation branch

The High Transformation Branch (HTB) represents the final stage of our network’s feature transformation hierarchy, where the focus shifts to synthesizing the final high-resolution image. This branch is architecturally similar to the Middle Transformation Branch (MTB) but is streamlined to contain lesser residual blocks, reflecting its specialized role in fine-tuning the feature maps for high-resolution output ($\hat{I}_H$).

The HTB receives the 2x upsampled output from the MTB ($\hat{I}_M$), which carries a rich blend of structural and spatial information. The upsampled feature map enters a convolutional layer that meticulously processes the enlarged feature set. Following this, a leaky ReLU activation function is applied.

The residual blocks at the core of the HTB are critical for refining the upsampled features. The reduced number of blocks compared to the MTB reflects the HTB’s role in applying the finishing touches to the image rather than transforming the feature set extensively. The initial convolutional layer of the HTB outputs only a 12-channel feature map to avoid the high computational complexity in the form of more GFLOPs, which arises as a result of the HTB having to operate on high-resolution features.

After the residual blocks, the feature map undergoes a final convolutional, consolidating the high-level features into a coherent image structure. This final convolutional layer is pivotal in rendering the fine details and ensuring that the synthesized high-resolution image retains the textural and structural integrity of the preceding branches. This output is added to L1, and a tanh activation is applied. The output of the HTB ($\hat{I}_H$) is the generation of the top layer of the translated pyramid. This image embodies the detailed textural information from the LTB and integrates the abstract structural and spatial features refined through the MTB, resulting in a comprehensive and high-fidelity visual output.

7 $$\begin{aligned} \hat{I}_H = \text {HTB}(I_{H}) \end{aligned}$$

where $\hat{I}_H$ represents the output of the HTB and $I_{H}$ is the input to the HTB.

8 $$\begin{aligned} \text {HTB}(x) = \tanh (\text {Conv}(f_{\text {res}}^K(\text {LeakyReLU}(\text {Conv}(x))))) \end{aligned}$$

where *x* represents the input feature map, $\text {Conv}$ denotes convolutional operations and $f_{\text {res}}^K$ represents *K* residual blocks.

### Inverse Laplacian reconstruction module

To ensure applicability to real-time problems and a lightweight architecture, we use bicubic interpolation to reconstruct the final image from the translated pyramid. The layers ($\hat{I}_L$), ($\hat{I}_M$), and ($\hat{I}_H$) are upsampled and added, in a cascading, inverse-laplacian operation, to reconstruct the final high-resolution thermal image in a non-parametric manner. The final reconstructed image is treated as our model’s prediction ($\hat{y}$) for the computation of the loss. This inverse operation eliminates transpose convolutions, thereby avoiding the need for more parameters.

### Discriminator

The discriminator serves as a crucial component for enhancing the perceptual quality of generated thermal images. We implemented a lightweight yet effective discriminator architecture that progressively captures hierarchical features while maintaining computational efficiency.

The discriminator begins with an initial convolutional layer that transforms the input thermal image into a 16-channel feature map, applying instance normalization to stabilize the training process. Following this, we implement a series of discriminator blocks that systematically downsample the spatial dimensions while expanding the feature depth (16; 32; 64; 128; 128). Each block enhances the network’s ability to detect increasingly complex patterns and structural details in the thermal images.

This progressive architecture allows the discriminator to effectively distinguish between authentic high-resolution thermal images and those generated by the generator described in earlier sections. The final layers of the network consolidate these learned features into a scalar judgment. This design complements our LSGAN adversarial loss implementation mentioned in Section 3.6, working in tandem with pixel-wise MSE loss to ensure faithful reconstruction of fine structural and spatial details including illumination, texture, and contrast.

By balancing discrimination capability with parameter efficiency, our discriminator reinforces LapGSR’s core philosophy of achieving high-quality thermal super-resolution while maintaining a lightweight computational footprint.

### Loss function

To ensure the ideal qualitative and quantitative quality of the final high-resolution thermal image ($\hat{I}_H$), we introduce a combined loss function to combat the tradeoff between PSNR and SSIM. Our loss function involves pixel-wise $\lambda$-weighted Mean Squared Error Loss (MSELoss) and Adversarial Loss.

**Mean Squared Error Loss** or MSELoss^23^ is the pixel-wise difference between the squares of the ground truth and the prediction, divided by the number of samples. This minimization allows for pixel-wise similarity between the ground truth and prediction.

**Adversarial Loss** is used to retain visual fidelity through an edge-guided network, we employ adversarial loss or GANLoss. Specifically, we employ a Least-Square Generative Adversarial Network (LSGAN)^24^ along with pixel-wise MSE to faithfully reconstruct fine structural and spatial details like illumination, texture, contrast, etc. The output of our model acts as a generator and tries to ’fool’ the discriminator. The equations for the Discriminator and Generator are given below:

9 $$\begin{aligned} L_{G} = \frac{1}{2} \mathbb {E}_{z \sim p(z)} \left[ (D(G(z)) - 1)^2\right] \end{aligned}$$

Where $G$ is the generator, $D$ is the discriminator, $z$ is a random noise vector sampled in a Gaussian distribution, and $D(G(z))$ is the discriminator’s prediction for the fake data^25^ produced by the generator.

10 $$\begin{aligned} L_{D} = \frac{1}{2} \mathbb {E}_{x \sim p_{\text {data}}(x)} \left[ (D(x) - 1)^2\right] + \frac{1}{2} \mathbb {E}_{z \sim p(z)} \left[ D(G(z))^2\right] \end{aligned}$$

Where $x$ is real data^25^, and $D(x)$ is the discriminator’s prediction for the real data. The **Final Loss** involves the weighted addition of MSELoss (reconstruction loss) and GANLoss (adversarial loss) with a balancing hyperparameter $\lambda$. This final loss ensures perceptual quality as well as spatial reconstruction. Hence, the final formula of the loss used in the model is:

11 $$\begin{aligned} L = \lambda L_{\text {MSELoss}} + L_{\text {GANLoss}} \end{aligned}$$

## Experiments

We conduct experiments for high-resolution thermal image generation on two datasets, namely ULB17-VT^4^ and VGTSR^6^. While VGTSR has slight misalignment between the RGB-Thermal pair, we also introduce external misalignment on various scales in the ULB17-VT dataset to additionally evaluate our model’s robustness.

### Datasets

**ULB17-VT**^4^ dataset contains mostly well-aligned, low-resolution thermal, high-resolution RGB, and high-resolution thermal images. The authors used a FLIR-E60 camera to capture thermal-visual images with a resolution of 320 x 240 pixels, a thermal sensitivity of 0.05°C, and a range of -20°C to 650°C. The published benchmark has 404 pairs of images divided into 280 training samples, 78 validation samples, and 46 testing samples. Since the low-resolution thermal image is 1 channel, the input RGB image is grayscaled. It contains scenes of various environments, such as indoor, outdoor, winter, and summer, of static and moving objects. We also experimented by introducing misalignment between the RGB and high-resolution thermal images. Table 3 demonstrates our results at different scales of misalignment.

**VGTSR**^6^, the dataset consists of 1025 pairs of visible, high-resolution, and low-resolution thermal images captured under UAV (Unmanned Aerial Vehicle) platforms. Each image has a resolution of 640 $\times$ 512 and contains a great number of small objects which are manually aligned. The thermal images have a resolution of 160 x 128 and have 3 channels. Hence, the input RGB image is considered a 3-channel image. The visible and thermal UAV images are taken by a DJI Matrice M300 RTK UAV equipped with the Zenmuse H20T sensor, which integrates multiple sensors to capture visible and thermal image pairs simultaneously. The authors of the dataset created the low-resolution images by bicubic and bilinear interpolation. To maintain synonymity with the ULB17-VT, we use the low-resolution thermal images created by bicubic interpolation.

Our choice of datasets further emphasizes the real-world application of our model. The ULB17-VT dataset is captured by a handheld camera, whereas the VGTSR dataset is captured by a UAV (Unmanned Aerial Vehicle) platform. Our model’s ability to maintain results in this cross-domain scenario allows for applicability in actual scenarios.

### Training setup

Through exhaustive experimentation, we decided upon our model’s best and most efficient configuration. As mentioned in Section 3.6, our combined loss consisted of a $\lambda$-scaled MSELoss and GANLoss. Given in Table 6 are our results of varying values of $\lambda$. The low-resolution thermal and high-resolution RGB images are normalized to a range of [0, 1]. Augmentation is performed on the dataset with vertical and horizontal flipping with a probability of 0.5. We have patched our images, like the authors of the dataset^6^, to alleviate computational load to a size of 40 x 30.

Another critical component of our model is the configuration of residual blocks in the LTB, MTB, and HTB. These residual blocks dictate the degree of feature extraction and transformation each branch enables. Hence, we extensively experimented with different numbers of residual blocks in each branch, as displayed in Tables 4 and 5. Our model utilized the Adam Optimizer^26^ and was trained for 100 epochs on the ULB17-VT dataset with a learning rate of $10^{-4}$ and batch size 12 on NVIDIA Tesla P100 GPU, and for 600 epochs on the VGTSR dataset with a learning rate of 3 x $10^{-2}$ on NVIDIA RTX A6000 due the large size of the dataset.

To evaluate our model’s results, we use the metrics Peak Signal Noise Ratio (PSNR)^27^, a quantitative measure defined by the ratio of the signal’s maximum power of the signal to the power of residual errors and Structural Similarity Index Measure (SSIM)^28^, a quantitative measure of spatial reconstruction in image generation. SSIM quantifies the perceptual quality of the generated image.

## Results

**Table 1:** Comparison of various GTSR methods against our model on the ULB17-VT dataset.

Method	#Params	PSNR	SSIM%
ZSSR^29^	-	26.79	85.67
VTSRGAN^17^	758K	27.99	82.02
DeepJF^30^	-	27.04	84.10
VTSRCNN^4^	758K	27.97	81.96
CMSR^20^	-	29.93	88.20
MMSR^22^	-	30.44	89.84
CMPNet^31^	2.02M	31.58	90.56
**LapGSR**	**398K**	**37.52**	**95.04**

Our model demonstrates state-of-the-art results with significantly fewer parameters. There are spaces left blank because the code for these repositories is not publically available for the calculation of parameters.

**Table 2:** Comparison of state-of-the-art super-resolution methods against our model on the VGTSR dataset.

Method	#Params	PSNR	SSIM%
Restormer^32^	25.3M	30.71	89.33
MultiNet^33^	8.7M	30.31	88.72
PAG-SR^21^	7.6M	30.74	89.51
UGSR^18^	4.5M	30.29	88.72
MGNet^6^	18.6M	31.16	90.33
CENet^34^	-	31.24	90.35
**LapGSR**	**773K**	**28.9**	**88.78**

In this study, we draw comparisons on the ULB17-VT dataset with various guided TSR models. In our experiments with various residual blocks in the lower, middle, and upper transformation branches, we discovered that for the ULB17-VT dataset, a configuration of 2, 3, and 3 residual blocks, respectively, achieves a PSNR of 37.024 and an SSIM of 0.9508 and for the VGTSR dataset, a configuration of 3, 5, and 7 residual blocks, respectively achieves a PSNR of 28.9 and SSIM of 0.887.

**Table 3:** Shift scale refers to the fraction by which the RGB image is shifted compared to the high-resolution thermal image.

Shift Scale	PSNR	SSIM%
0.05	35.17	95.53
0.1	36.95	95.59
0.15	34.96	95.80

If shift_limit is a float, the range will be (-shift_limit, shift_limit).

We present the exceptional outcomes of LapGSR as shown in Table 1 on the aligned ULB17-VT dataset. Our model not only achieves SOTA results but also remarkably reduces parameter usage to 398K, outlining its efficiency and real-world application. We compare our model with competing SR methods such as a generative adversarial network (VTSRGAN), a CNN-based network (VTSRCNN)^4^, and a joint cross-modality transformer (CMSR)^20^ for the task of guided thermal super-resolution and achieve SOTA results on the ULB17-VT dataset.

**Fig. 3:**
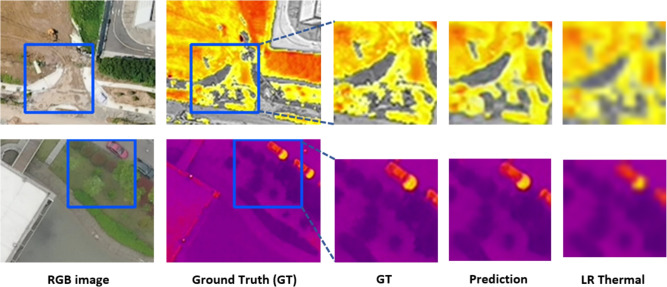
Visualization of our model’s output on two instances of the VGTSR dataset. The first image in each row is the high-resolution RGB image, the second is the ground truth, the third is a patch of the Ground Truth, the fourth is a patch of LapGSR’s output, and the last is a patch of the low-resolution thermal image.

Extending our analysis to the VGTSR dataset, our model attains comparable results as shown in Table 2, confirming its prowess in generating high-resolution thermal images. As mentioned by the authors of the dataset in^6^, UAV images have a larger range of observation due to the shooting height. This leads to UAV images containing more contour information and less texture information (Fig. 3). Since our model relies on explicit edge-guidance, the lack of textural information leads to slightly diminished performance. Our work illustrates that our approach, while slightly underperforming in terms of PSNR (2.34) and SSIM (0.157) significantly reduces the computational complexity by cutting down the parameter count by around 95%.

We establish our model’s robustness by evaluating performance on cases of misalignment, primarily on the ULB17-VT dataset, demonstrating our model’s ability to maintain consistent super-resolution quality (Table 3). This robustness is facilitated by augmentations that simulate real-world shifts in perspective. Marginal non-linearity in the trends of results are observed in Table 3 due to our adopted random shift scaling method. As the caption mentions, random shift scaling shifts every image by any value between (-shift_limit, shift_limit). Since there can be a variance in shifting, it may produce a slight variance (about 5%) in results.

As evident in Fig. 3, our results are visually apt and similar to the ground truth. The visualization of two instances is given row-wise. The first image of each row is the RGB image, the second is the Ground Truth, the third is the patch from the Ground Truth, the fourth is the patch of LapGSR’s output, and the last is the low-resolution thermal. On observing the 1st row, it is evident that the RGB image lacks textural information, which results in our model’s output being ’smooth’ and lacking visual fidelity. The images in the 2nd row contain rich textural information, which results in a highly accurate prediction.

## Qualitative results

**Fig. 4:**
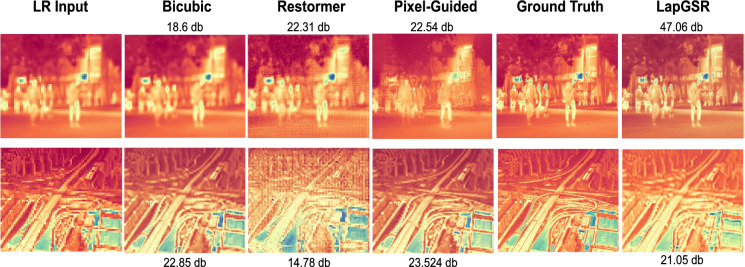
Comparison of LapGSR’s outputs with existing methods: Restormer^32^, and Pixel-Guided SR method PixTransform^35^. The first row contains an instance from the ULB17-VT dataset and the second row contains an instance from the VGTSR dataset. The PSNR score of each image is mentioned in Decibels (db).

Figure 4 compares the performance of LapGSR on instances from both datasets against SOTA methods like Restormer^32^, and Guided SR methods like PixTransform^35^ and a standard Bicubic implementation. From the PSNR scores and qualitative analysis, it is evident that LapGSR reconstructs images of the ULB17-VT dataset with impressive ability while accurately predicting the pixel intensities and gradients. Besides, a significant improvement is observed in the reconstructed image using LapGSR as compared to Restormer^32^. However, as evident in the second row of the same figure, the model slightly underperforms with respect to PixTransform^35^, where the intensity of the pixel is inconsistent and the image quality is slightly sub-par, leading to only a comparable reconstruction.

**Fig. 5:**
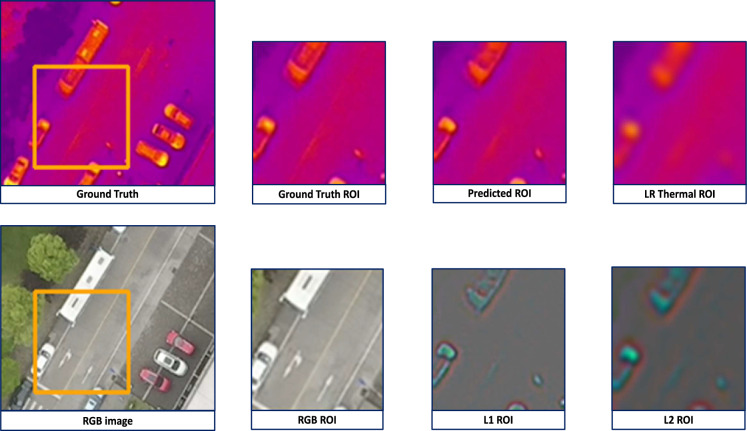
This is an instance from the VGTSR dataset. The highlighted region of interest (ROI) in the RGB image has sharp edges for the car and bus. This is also present in the ROI of the subsequent Laplacian pyramid, which helps our edge-guided model learn accurate representations of the thermal image, as evident in the predicted ROI. L1 and L2 stand for the first and second layers of the Laplacian Pyramid.

Notably, our model accurately reconstructs thermal images when the ’guide’ RGB image contains adequate textural information, as demonstrated by Figs. 5 and 7. For each instance, the low-resolution thermal region of interest has been bilinearly upsampled for clear visualization.

**Fig. 6:**
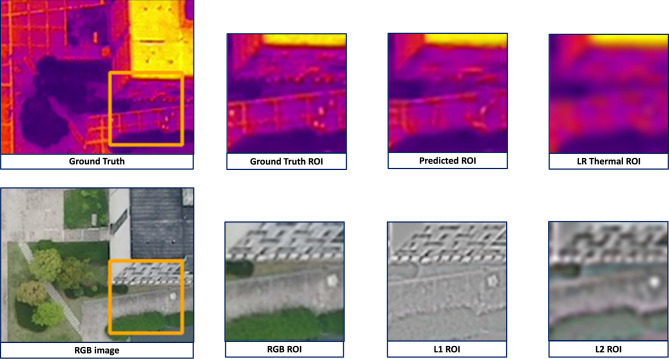
This is a patch from an instance of the VGTSR dataset. The highlighted region of interest (ROI) in the RGB image has blurry driveway edges. This lack of textural information is also present in the ROI of the subsequent Laplacian pyramid, which results in sub-par results of our model, evident in the Predicted ROI. L1 and L2 stand for the first and second layers of the Laplacian Pyramid.

Figure 5 is an instance from the VGTSR dataset. In this case, the RGB image contains clear edge information, as depicted in its Laplacian layers, L1 and L2. This allows the model to learn features and accurately reconstruct a high-resolution thermal image. However, Fig. 6 contains more contour information and less textural information. In Fig. 6, an instance of the VGTSR dataset, the exact details of the road/driveway are missing in the RGB image and the L1 and L2 layers of its Laplacian pyramid. This leads to these details being absent in our model’s prediction. Figure 5, an instance of the VGTSR dataset, contains sharp details reflected in the RGB image’s Laplacian pyramids, which allows our model to predict accurate and visually apt results.

**Fig. 7:**
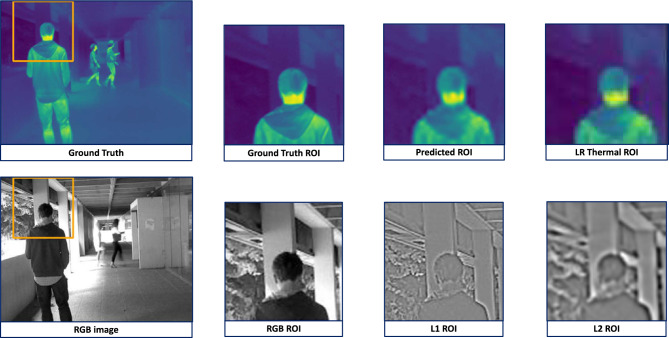
This is a patch from an instance of the ULB17-VT dataset. The highlighted region-of-interest (ROI) in the RGB image has precise edges of the man. This abundance of textural information is also present in the ROI of the subsequent Laplacian pyramid, which results in excellent results for our model, as evident in the Predicted ROI. L1 and L2 stand for the first and second layers of the Laplacian Pyramid.

**Fig. 8:**
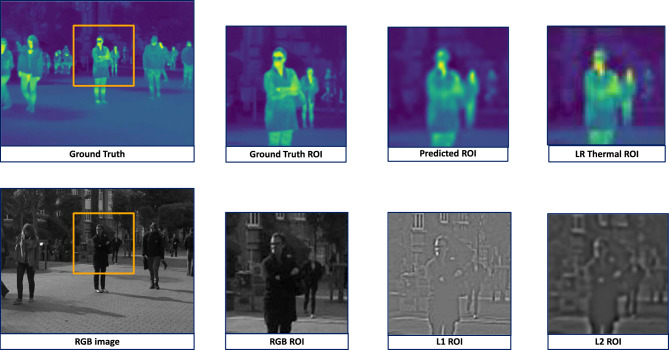
This is a patch from an instance of the ULB17-VT dataset. The highlighted region of interest (ROI) in the RGB image has blurred edges of the person’s arms and spectacles. This consistency of texture results in poor edge information, as visualized in the ROI of the Laplacian Pyramids. Hence, this results in slightly compromised results, as evident in the Predicted ROI. L1 and L2 stand for the first and second layers of the Laplacian Pyramid.

Figure 7, taken from the ULB17-VT dataset, contains adequate textural enough to allow our model to capture finer details and reproduce a visually appealing image. The RGB images in both figures contain explicit edge details, evident in the Laplacian pyramid’s layers, allowing our model to generate more realistic and accurate outputs. Figure 8, also taken from the ULB17-VT dataset, demonstrates subpar results. This is because, on observing the RGB image, it is noted that no edges are present on the man’s torso in the ROI. This results in its Laplacian pyramid’s layers also containing minimal edge information, leading to our model being unable to capture finer details.

Hence, it is evident from all our examples that instances where edge information is subpar lead to only passable results for our model. Although these images are seldom present in the ULB17-VT dataset, they are ubiquitous in the VGTSR dataset.

## Ablation study

In this section, we experimentally study the effects of different parameters and modules on the results of our super-resolution method. We run these experiments on the ULB17-VT and VGTSR datasets. These extensive experiments allow us to pick our model’s best configuration of residual blocks and the most balanced loss weight. Although our results on the VGTSR dataset are shown on 600 epochs, we conducted ablations with 400 epochs due to the large computational load.

**Table 4:** Our results on the ULB17-VT dataset with several configurations of residual blocks.

Residual Blocks			Params	GFPs	Metrics
LTB	MTB	HTB			PSNR/SSIM%
3	2	3	398K	9.34	35.88/94.97
3	3	3	471K	12.18	36.50/93.99
3	4	3	546K	15.02	39.72/94.44
3	5	3	620K	17.86	34.71/95.31
4	3	3	546K	12.9	36.24/95.95
5	3	3	620K	13.6	39.68/94.38
3	3	2	469K	11.78	35.98/95.19
3	3	4	475K	12.58	35.06/94.34
3	3	5	477K	13	39.67/94.49
**2**	**3**	**3**	**398K**	**5.74**	**37.52**/**95.04**

GFPs stands for GigaFLOPs.

In Table 4, we present our results on the ULB17-VT dataset with various configurations of residual blocks with $\lambda$ value as 4500. Through exhaustive experimentation, we observed that the number of residual blocks in the LTB, MTB, and HTB of 2, 3, and 3 demonstrates the best results. A tradeoff exists between the PSNR and SSIM, which is evident when the total number of residual blocks increases, causing either metric to increase at the cost of the other. Moreover, the number of trainable parameters increases beyond the configuration of 2, 3, and 3 while not enhancing performance.

**Table 5:** Our results on the VGTSR dataset with several configurations of residual blocks.

Residual Blocks			Params	GFPs	Metrics
LTB	MTB	HTB			PSNR/SSIM%
3	3	3	478K	3.32	27.68/85.94
3	5	3	626K	4.84	28.04/87.78
3	7	3	773K	6.36	28.33/88.24
3	9	3	921K	7.86	28.31/88.16
3	7	1	768K	6.14	28.18/88.14
1	7	3	626K	5.98	28.20/87.79
5	7	3	921K	6.74	28.24/88.27
7	7	3	1.07M	7.10	28.37/88.40
**3**	**7**	**5**	**779K**	**6.56**	**28.35/88.08**

GFPs stands for GigaFLOPs.

Directing our attention to the VGTSR dataset, we adopted a similar strategy of exhaustive experimentation for this dataset. We note that the best configuration of the number of residual blocks in the LTB, MTB, and HTB, respectively, are 3, 7, and 5 with $\lambda$ of 4500. An increase in the number of blocks causes a negligible change in performance at the cost of a high number of parameters. As evident in Table 5, the number of residual blocks needed for comparable results is more than for the ULB17-VT dataset. This is because the images in the VGTSR dataset are more complex and have more contour than textural information.

**Table 6:** Our results on the ULB17-VT dataset with various values of $\lambda$.

Loss Weight ($\lambda$)	PSNR	SSIM%
0	12.93	81.85
1500	36.51	94.91
2500	36.03	94.95
3500	37.05	95.11
**4500**	**37.52**	**95.04**
5500	37.42	95.12
6500	37.46	95.22

As mentioned in Section 3.6, we introduce a combined loss, merging GANLoss and $\lambda$-weighted MSELoss. As evident in Table 6, we conducted thorough experimentation with several values of $\lambda$ on the ULB17-VT dataset. We observe that increasing $\lambda$ to 4500 demonstrates the best results, and increasing it further causes negligible changes in the final PSNR and SSIM.

In order to obtain the best results, we conducted several experiments with various Adversarial Losses, as mentioned in Table 7. As evident, the LSGAN demonstrates the best results with the given configuration of residual blocks (2, 3, 3 for ULB17-VT and 3, 7, 5 for VGTSR) and a loss weight of 4500. Hence, we implement the LSGAN Loss in tandem with MSELoss to obtain state-of-the-art results.

**Table 7:** The results of our model with various GAN loss functions.

GAN Type	ULB17-VT^36^	VGTSR^6^
	PSNR/SSIM%	PSNR/SSIM%
Vanilla GAN^37^	36.47/94.82	28.05/88
WGAN^38^	36.94/94.89	27.81/87.44
Hinge GAN^39^	36.74/94.86	28.17/87.79
**LSGAN**^24^	**37.52**/**95.04**	**28.9**/**88.78**

As mentioned in the results section, the best configurations of Residual Blocks are used in the LTB, MTB and HTB for each dataset, respectively along with the best loss weight.

## Conclusion and future works

We propose LapGSR, a lightweight model that uses Laplacian image pyramids to improve the resolution of thermal images. The model decomposes the original RGB image into a modified Laplacian pyramid, which preserves finer image details. This bypasses the need for heavy, high-resolution feature map computation, resulting in a condensed and efficient model. LapGSR can produce SOTA results while being robust and lightweight (398 K parameters on the ULB17-VT dataset and 773 K on the VGTSR dataset), making it suitable for real-world applications.

One of the limitations of our proposed method is its performance with aerial view images. This is because our model uses a non-parametric Laplacian reconstruction module to generate the final thermal image. This module cannot account for the loss of edge information that occurs in RGB images when the objects are at a distance. In the future, we plan to address this limitation by making provisions for feature extraction from high-altitude RGB images. This would allow the architecture to better handle UAV images and produce more accurate super-resolved thermal images. We plan to make our codebase open-source upon the acceptance of our paper.

## Data Availability

The work utilized publicly available standard datasets, namely ULB17-VT and VGTSR. These two datasets are available at https://github.com/uverma/LapGSR.
